# Cost-utility analysis of a polylactic acid matrix versus a collagen dressing for the closure of diabetic foot ulcers

**DOI:** 10.3389/fpubh.2025.1625252

**Published:** 2025-10-13

**Authors:** Elaheh Khorasani, Aashita Batra, Robert Bartlett, Stephen Bergquist, Brock A. Liden, Karla Rangel-Berridi

**Affiliations:** ^1^Division of Experimental Surgery, McGill University, Montreal, QC, Canada; ^2^Independent Scientist, Boston, MA, United States; ^3^CutisCare, Boca Raton, FL, United States; ^4^Wound Management Center, The Jackson Clinic, Jackson, TN, United States; ^5^WAFL, Circleville, OH, United States

**Keywords:** diabetic foot ulcer, polylactic acid matrix, cost-effectiveness, randomized controlled trial, quality-adjusted life years, economic burden

## Abstract

**Background:**

Diabetic foot ulcers (DFUs) are a common and serious complication of diabetes, often requiring the use of advanced care products, which may have high upfront costs. With the hypothesis that this approach leads to faster healing, reduced costs, and better quality-adjusted life years (QALY), this study evaluates the economic outcomes, including cost-effectiveness and cost–benefit, of an alloplastic polylactic acid (PLA) dermal matrix and compares it to collagen dressings in managing DFUs.

**Methods:**

This cost-utility analysis was based on a randomized controlled trial involving patients with DFUs treated with either PLA matrices or collagen dressings, alongside standard wound care. Data on wound healing, cost of care, and QALY were collected over the whole duration of the trial (31-week period). We conducted a cost–benefit analysis by quantifying the monetary impact of reduced time-to-heal and avoided healthcare utilization. Additionally, we performed a cost-utility analysis using QALYs to capture patient-centered benefits.

**Results:**

At 12 weeks, 90% of the PLA group achieved wound closure compared to 30% in the collagen group, with PLA matrices reducing healing time by 44%. The cumulative cost of treatment for PLA was significantly lower, averaging $2,928 compared to $5,542 for collagen dressings (*p* < 0.001). Sensitivity analyses confirmed the cost-effectiveness of PLA even when home healthcare costs were excluded. Cost–benefit analysis also demonstrated higher QALY in the PLA treated group.

**Conclusion:**

PLA dermal matrices provide a cost-effective alternative to collagen dressings, promoting faster wound closure, improved quality of life, and reduced healthcare costs. These results support the adoption of PLA as a preferred treatment for DFUs.

## 1 Introduction

Diabetic foot ulcers (DFUs) are a significant and debilitating complication of diabetes, affecting up to 35% of individuals with the disease during their lifetime (1, 2). These ulcers often develop as a result of peripheral neuropathy, ischemia, and poor glycemic control, creating an environment in which wounds become chronic and are difficult to heal (3). The inability to effectively treat DFUs can lead to severe outcomes, such as infection, gangrene, and lower-extremity amputations, with DFUs contributing to approximately 85% of diabetes-related amputations (4). Moreover, the five-year mortality rate following an amputation is alarmingly high, with rates exceeding 70%, further underscoring the critical need for more effective DFU treatments (3).

Beyond their clinical impact, DFUs impose a substantial economic burden on healthcare systems worldwide. In the United States alone, the annual cost of treating DFUs was estimated to range from $9 billion to $13 billion in the last decade (1, 5). However, more recent estimates of acute and chronic wound treatments range from $28 billion to $96 billion (6). This disparity probably reflects methodological differences in how costs were calculated and real-world trends, including the rising cost of living and medical services, the expanded use of advanced wound care products, and the increased burden of complications such as infection and limb salvage surgeries. Together, these factors underscore the escalating economic impact of diabetic foot ulcers and generate economic strain steming from extended healing times, frequent medical interventions, and the need for long-term care, including home healthcare and specialized wound management services (5, 7). Traditional wound care approaches, such as collagen-based dressings, have been associated with limited efficacy, with fewer than 50% of DFUs achieving complete closure after 12 weeks of treatment (8). Thus, wound care represents a major clinical, social, and economic challenge, with expenditures related to wound care ever mounting and far exceeding historical estimates. This economic burden highlights the need for innovative and cost-effective therapies that can accelerate healing, reduce complications, and improve patient outcomes.

The current management of DFUs typically includes standard wound care measures such as debridement, infection control, moist wound healing, and offloading. Advanced wound care products, including collagen dressings and cellular, acellular, and matrix-like products (CAMPs), are widely used to support healing when standard care is insufficient (3, 9, 10). Among the new generation CAMPs, alloplastic polylactic acid (PLA) dermal matrices have shown excellent potential in promoting the closure of DFUs (11, 12). PLA acts by releasing lactate as a by-product of its degradation, which serves as a potent signaling molecule with several key biological functions. At the cellular level, lactate stabilizes hypoxia-inducible factor-1α (HIF-1α), driving VEGF expression and stimulating neo-angiogenesis. It provides a preferential oxidative substrate that supports fibroblast and keratinocyte survival and proliferation, while also modulating macrophage polarization from pro-inflammatory (M1) to reparative (M2) phenotypes, thereby reducing inflammation. In addition, lactate lowers wound bed pH, creating an environment less favorable for bacterial growth and more conducive to enzymatic processes of healing. Collectively, these mechanisms explain the superior biological performance of PLA membranes and provide the foundation for the improved clinical outcomes that underpin our health economic evaluation (13). A randomized controlled trial (RCT) comparing PLA matrices to collagen dressings highlighted their superior ability to accelerate wound healing (11). In addition to standard care (debridement, wound care, and offloading with a walking boot), patients treated with PLA matrices experienced a 44% reduction in time to heal compared to those receiving collagen dressings. By the 12th week, 90% of wounds treated with PLA matrices had fully healed, compared to only 30% in the collagen group (11). This significant reduction in healing time positions PLA matrices as a valuable therapeutic option for DFUs, especially in cases where conventional therapies have failed to produce satisfactory outcomes.

Given the significant healthcare burden posed by DFUs, cost-effectiveness analysis is essential in evaluating new treatments. Developing wound care products that are both clinically effective and economically viable is critical to reducing the strain on healthcare systems (6). Advanced wound care technologies, such as PLA matrices, offer the potential to not only improve clinical outcomes but also reduce the need for prolonged care and associated costs (5). Therefore, the objective of this study is to analyze the economic data from the RCT (11) and perform a cost-utility analysis to highlight both the immediate economic advantages and the health outcomes of using PLA wound closure matrices in managing DFUs. By combining real-world cost data, including expenses related to debridement, wound care, and home nursing visits, with health outcomes measured in quality-adjusted life years (QALY), this analysis seeks to determine whether the higher upfront costs of PLA matrices are justified by their ability to improve patient outcomes and reduce long-term healthcare expenditures. These findings will help inform clinical decisions and optimize resource allocation in DFU treatment.

## 2 Materials and methods

### 2.1 Study design and compliance

The primary data for this analysis was sourced from the RCT conducted by Liden and Ramirez-Garcialuna in 2023 (11). This study compared the healing outcomes of standard of care wound care plus either PLA matrices or collagen dressings over a 28-week period in a sample of 30 patients (15 per arm). The trial was registered with ClinicalTrials.gov (NCT05883098). The trial was approved by WCG Clinical IRB (Protocol Number 20230304). The studies were conducted in accordance with the local legislation and institutional requirements. The participants provided their written informed consent to participate in this study. The analysis followed ethical standards, and all participants provided informed consent prior to enrollment. It followed a parallel-group design where patients with DFUs were randomly assigned to receive either weekly applications of PLA matrices (Supra SDRM, Polymedics Innovations, Denkendorf, Germany) or collagen dressings (Fibracol Plus, 3M) until healing, in addition to standard care consisting of debridement, wound care, and offloading with a walking boot. Although it is not considered a CAMP, collagen dressings have widely been considered the clinical standard of care for advanced wound management and are traditionally the comparison standard in most clinical trials (14). Inclusion criteria included a single Wagner grade 1 or 2 DFU with a duration of 12 weeks to 12 months and a size of 1 to 25 cm^2^. Patients were required to have an HbA1c < 10% and an ankle-brachial index (ABI) between 0.7 and 1.3 within 2 months of randomization. Offloading of the ulcer for a minimum of 14 days before randomization with a size reduction of <20% was also required. Exclusion criteria included active wound infections, gross edema, uncontrolled comorbidities, or the use of drugs that would modify wound healing. Elimination criteria included the development of infections requiring systemic antibiotics and the loss to follow-up for two or more consecutive visits.

The Liden trial aimed to evaluate the healing time, cost of care, and health outcomes in both groups over the duration of the study. Effectiveness data, including weekly wound size measurements, were collected from the RCT and used as the primary input for the cost-utility analysis. Reporting of the data was done following the Consolidated Health Economic Evaluation Reporting Standards (CHEERS) guideline (15) from the Enhancing the QUAlity and Transparency Of health Research (EQUATOR) Network.

### 2.2 Effectiveness data

The effectiveness data were derived from the Liden 2023 RCT, which collected information on patient demographics, wound characteristics, and clinical outcomes. The patients in the trial were treated for DFUs, and the primary data collected included clinical characteristics relevant to wound healing. Weekly wound measurements were taken to monitor wound area reduction and the time to full healing was recorded. The primary endpoint was the number of weeks until full wound closure, while secondary endpoints included the percentage of wound area reduction and total costs incurred during the treatment period. The patients’ characteristics and main outcomes are presented in Table 1. Baseline patient demographics and wound characteristics were comparable between the two groups, with no significant differences observed. The only significant differences observed were in the outcomes of interest, time to heal and healing rates at 12 weeks, which were superior in the PLA group.

**Table 1:** Patient and wound characteristics.

Variable	Collagen (*N* = 15)	PLA (*N* = 15)	*p*-value
Age (years)			0.972
Mean (SD)	63.8 (14.5)	64.0 (10.5)	
Gender			0.143
Female	6 (40.0%)	5 (34.0%)	
Male	9 (60.0%)	10 (66.0%)	
HbA1c (%)			0.200
Mean (SD)	7.6 (0.9)	8.2 (1.2)	
ABI			0.417
Mean (SD)	1.05 (0.12)	0.99 (0.17)	
Ulcer Age (weeks)			0.636
Mean (SD)	15.4 (3.7)	16.8 (8.4)	
Area (cm^2^)			0.120
Mean (SD)	4.06 (2.19)	6.41 (3.98)	
Ulcer site			0.443
Dorsum	6 (40.0%)	6 (40.0%)	
Heel	2 (13.0%)	1 (6.0%)	
Metatarsal	3 (20.0%)	3 (20.0%)	
Plantar	4 (27.0%)	5 (34.0%)	
Time to Heal (weeks)			0.021
Median (SD)	14.8 (8.1)	9.3 (2.9)	
Wounds Healed by 12 Weeks (%)	5 (33.0%)	12 (80.0%)	0.025

### 2.3 Cost data collection

Cost data for this analysis were sourced from the Centers for Medicare & Medicaid Services Physician Fee Schedule for the State of Ohio, based on the 2022 cost listings, as the trial was done during this year. The direct medical costs included debridement for collagen dressings based on current procedural terminology (CPT) codes 11,042 and 11,045 or wound care for PLA matrices based on CPT code 15275, which includes the debridement procedure and the application of a CAMP. Walking boots under CPT code L4386 were used for both groups. These CPT codes correspond to the procedures routinely invoiced in the United States for debridement, wound care, and application of cellular- and tissue-based products. They are assigned and maintained by the American Medical Association and are enforced by insurance carriers and government payers such as Medicare. Their inclusion in the study ensures that the cost calculations accurately reflect real-world billing practices for both treatment arms. Additional wound care materials, such as non-adherent dressings (SUPRA Net/Rylon-1, Polymedics Innovations, Denkendorf, Germany), superabsorbent dressings (DryMax Extra, MPMMed, Mesquite, TX), and offloading devices (CAM walking boot), were also factored in, along with the costs of home nursing visits for wound care management. A detailed list of codes and costs associated are presented in Table 2. Two critical time points were used to assess costs: the 12-week mark, where most patients treated with PLA matrices had achieved wound closure, and the full healing time for patients in both the PLA matrices and collagen dressings treatment groups.

**Table 2:** Costs analyzed.

Healthcare services	Code (CPT)	Cost
PLA matrix (per cm^2^)	N/A	$59.69
Collagen dressing (per 4 cm^2^ piece)	N/A	$10
DryMax superabsorbent dressing	N/A	$10
SUPRA Net/Rylon-1 non-adherent dressing	N/A	$13.20
Debridement (only Collagen group)	11,042	$126.07
Wound Care and CPT application (only PLA group)	15,275	$156.84
Walking Boot	L4386	$167.28
At-home nursing care (home health)	N/A	$177.53

A sensitivity analysis was conducted under two scenarios. In the first scenario, product costs were calculated using only the exact amount of materials required, without accounting for any wastage. In the second scenario, product usage was rounded up to account for waste, offering a more realistic estimate of total costs. These scenarios provided a complete understanding of the cost implications for each treatment option.

### 2.4 Health utility data and cost calculations

Utility values used in this analysis were sourced from literature and reflected the quality of life associated with healed and unhealed DFUs (16). A utility value of 0.80 was assigned to healed ulcers, while unhealed ulcers, defined as active but uninfected wounds, were assigned a utility value of 0.60, derived from previous studies (16, 17). These values, derived from diabetic patient populations, are considered a benchmark in DFU cost-utility modeling. Health outcomes were measured in terms of QALYs, which were calculated by multiplying the time spent in each health state (healed or unhealed) by the corresponding utility value. The total and mean costs of treatment for each group were calculated based on the direct medical costs incurred, including product costs, debridement, wound care, offloading devices, and home visits for nursing care. Similar to the cost analysis, two key time points were used to assess outcomes: the 12-week mark, when most PLA-treated patients had healed, and the full healing point, which extended to 31 weeks for patients treated with collagen dressings. A sensitivity analysis was conducted to assess the robustness of the results under different cost assumptions, including scenarios with and without home care costs, as well as with or without product waste, where any use of a piece was rounded up to the cost of the full unit. For example, if only half of a 5 × 5 cm piece was required, the proportional cost scenario would assign 12.5 cm^2^ of product, while the wastage scenario would assign the full 25 cm^2^ cost. This approach ensures that our sensitivity analysis accounts for the practical realities of clinical use and product pricing.

### 2.5 Statistical analysis

The primary analysis compared the total and mean costs between the PLA matrix and collagen dressing groups at both the 12-week and 31-week time points. Health outcomes were analyzed using QALYs, and the results were evaluated for both cost and effectiveness. QALYs were calculated by multiplying the time spent in a given health state by the corresponding utility value, consistent with standard methodology (18). As mentioned above, utility values were derived from published DFU-specific studies (16, 17), with healed ulcers assigned a value of 0.80 and unhealed ulcers a value of 0.60. For example, a patient with a healed ulcer after 12 weeks contributes 0.23 QALYs for the healed state (0.80 × 0.29 years) plus 0.09 QALYs for the unhealed state (0.60 × 0.17 years), yielding a total of 0.32 QALYs over 28 weeks. A sensitivity analysis was performed to explore the impact of varying key cost assumptions, such as the inclusion or exclusion of home care services and the rounding up of product usage for potential waste. Comparisons between groups were performed using ANOVA tests. ANOVA was selected to allow for potential extension of the analysis to more than two comparators in future studies. Normality of the distributions for outcomes was assessed using Shapiro–Wilk tests, which confirmed that parametric methods were appropriate. Accordingly, results are reported as mean ± standard deviation (SD). The analysis was conducted using the R v.4.4.2 statistical software (The R Core Team, 2024) at the 95% CI.

## 3 Results

Data from 30 patients (15 per treatment arm) was collected and analyzed. Table 3 presents the cost breakdown for the first 12 weeks of the trial, and Table 4, the cumulative costs for the same period.

**Table 3:** Mean weekly costs.

Time point	Collagen (*N* = 15)	PLA (*N* = 15)	*p*-value
Baseline			< 0.001
Mean (SD)	376.64 (5.53)	470.67 (87.54)	
Week 01			0.003
Mean (SD)	375.80 (5.42)	440.93 (71.67)	
Week 02			0.891
Mean (SD)	375.06 (5.28)	369.437 (157.429)	
Week 03			0.306
Mean (SD)	374.22 (4.95)	327.45 (173.72)	
Week 04			0.146
Mean (SD)	348.89 (96.62)	264.48 (195.88)	
Week 05			0.159
Mean (SD)	323.56 (131.42)	234.24 (199.44)	
Week 06			0.027
Mean (SD)	322.89 (131.15)	179.07 (198.82)	
Week 07			0.010
Mean (SD)	297.89 (154.22)	125.764 (184.21)	
Week 08			0.002
Mean (SD)	272.93 (170.39)	98.619 (169.29)	
Week 09			< 0.001
Mean (SD)	248.07 (181.59)	48.48 (127.93)	
Week 10			< 0.001
Mean (SD)	223.29 (188.74)	0.0 (0.0)	
Week 11			< 0.001
Mean (SD)	198.53 (192.24)	0.0 (0.0)	
Week 12			< 0.001
Mean (SD)	173.82 (192.35)	0.0 (0.0)	

**Table 4:** Weekly cumulative mean costs.

Time point	Collagen (*N* = 15)	PLA (*N* = 15)	*p*-value
Baseline			< 0.001
Mean (SD)	376.64 (5.53)	470.67 (87.54)	
Week 01			< 0.001
Mean (SD)	752.44 (10.94)	911.59 (158.79)	
Week 02			0.048
Mean (SD)	1,127.50 (16.18)	1,281.03 (287.37)	
Week 03			0.356
Mean (SD)	1,501.72 (21.06)	1,608.48 (440.09)	
Week 04			0.885
Mean (SD)	1,850.61 (107.51)	1,872.97 (585.94)	
Week 05			0.742
Mean (SD)	2,174.17 (222.69)	2,107.21 (747.66)	
Week 06			0.402
Mean (SD)	2,497.06 (349.25)	2,286.28 (893.56)	
Week 07			0.201
Mean (SD)	2,794.94 (478.22)	2,412.04 (1,026.45)	
Week 08			0.041
Mean (SD)	3,067.87 (616.53)	2,510.66 (1,153.90)	
Week 09			0.016
Mean (SD)	3,315.94 (761.18)	2,559.14 (1,210.39)	
Week 10			0.008
Mean (SD)	3,539.23 (909.46)	2,559.14 (1,210.39)	
Week 11			< 0.001
Mean (SD)	3,737.76 (1057.08)	2,559.14 (1,210.39)	
Week 12			< 0.001
Mean (SD)	3,911.58 (1201.71)	2,559.14 (1,210.39)	

### 3.1 Cost effectiveness with exact material usage without accounting for wastage

#### 3.1.1 Treatment cost over time

**Figure 1:**
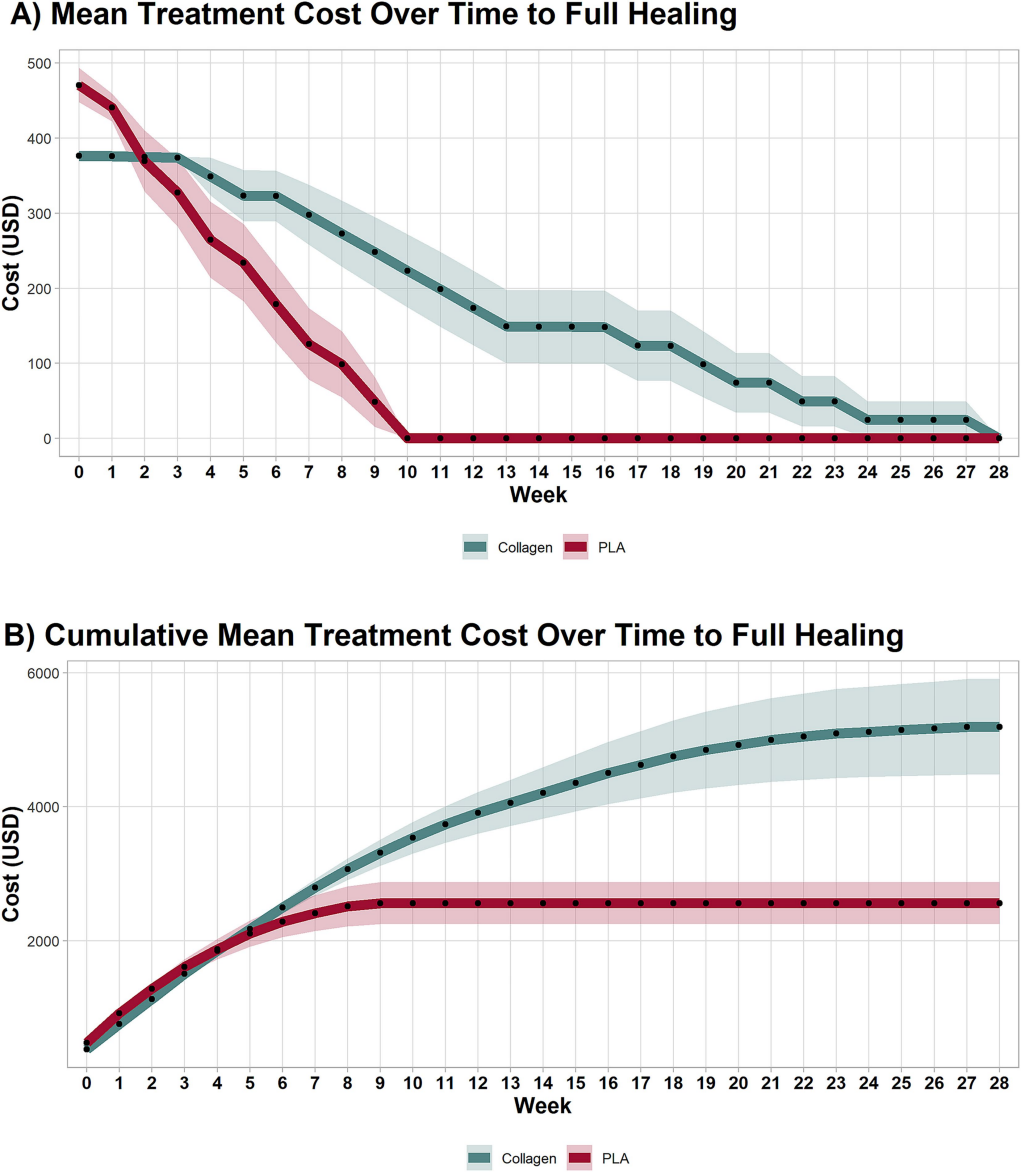
Cost of each treatment arm by week. **(A)** Mean treatment costs per week until healing. **(B)** Cumulative weekly mean treatment cost until healing. PLA intervention costs decreased rapidly, with no additional costs after the 10th week due to complete healing. Collagen treatment incurred costs up to the 28th week. PLA also improved patients’ quality of life in a shorter period than collagen. Shaded areas represent the 95% CI.

In this scenario, product costs were calculated based on the exact amount of material required, without accounting for wastage (i.e., price of advanced wound care products per square centimeter). As shown in Figure 1A, despite initially having a higher cost at baseline ($470.67 vs. $376.64, mean difference $94.03, 95% CI 47.64 to 140.40, *p* < 0.001), the treatment costs over time between the PLA and collagen groups equalized in week 2 (mean difference $5.62, 95% CI −88.93 to 77.69, *p* = 0.89). PLA treatment costs decreased rapidly, with no further costs incurred after week 10, as all patients in this group had healed. In contrast, the collagen group continued to accrue costs until week 28. This difference reflects the longer healing time required for the collagen-treated patients.

#### 3.1.2 Cumulative treatment costs

The cumulative treatment costs, presented in Figure 1B, demonstrate a marked cost difference between the two groups. By week 3, the cumulative costs for collagen-treated patients equalized those of PLA treated patients (mean difference $−106.8, 95% CI −126.30 to 339.81, *p* = 0.35), and by week 8, they had surpassed those of the PLA group (mean difference $557.00, 95% CI 134.71 to 1,249, *p* = 0.009). By the end of the study, the total mean cost per patient in the collagen group was nearly twice that of the PLA group. Specifically, the cost to achieve wound closure was $5,409.36 for collagen and $3,157.71 for PLA, representing a mean difference of $2,251.67 (95% CI 1,018 to 4,209, *p* < 0.001). The 95% confidence intervals provide an indication of the precision of the cost estimates. Narrower intervals, as observed for baseline costs, suggest higher precision, while wider intervals, as seen at later time points, reflect greater variability in patient-level outcomes due to the healing trajectories and the presence of a smaller available patient pool at later time points.

#### 3.1.3 Costs at 12 weeks

By the 12-week mark, 80% patients in the PLA group had already achieved full healing, compared to 30% in the collagen group (*p* < 0.001). The mean cost difference at 12 weeks between collagen and PLA was $173.80 (95% CI 72.08 to 275.60, *p* < 0.001), and the cumulative mean cost difference at 12 weeks was $1,352 (95% CI 450.30 to 2,225.00, *p* < 0.001). A sensitivity analysis that excluded home visit costs confirmed that the cost advantage of PLA at 12 weeks remained robust under various scenarios.

#### 3.1.4 Detailed breakdown of cost components

**Figure 2:**
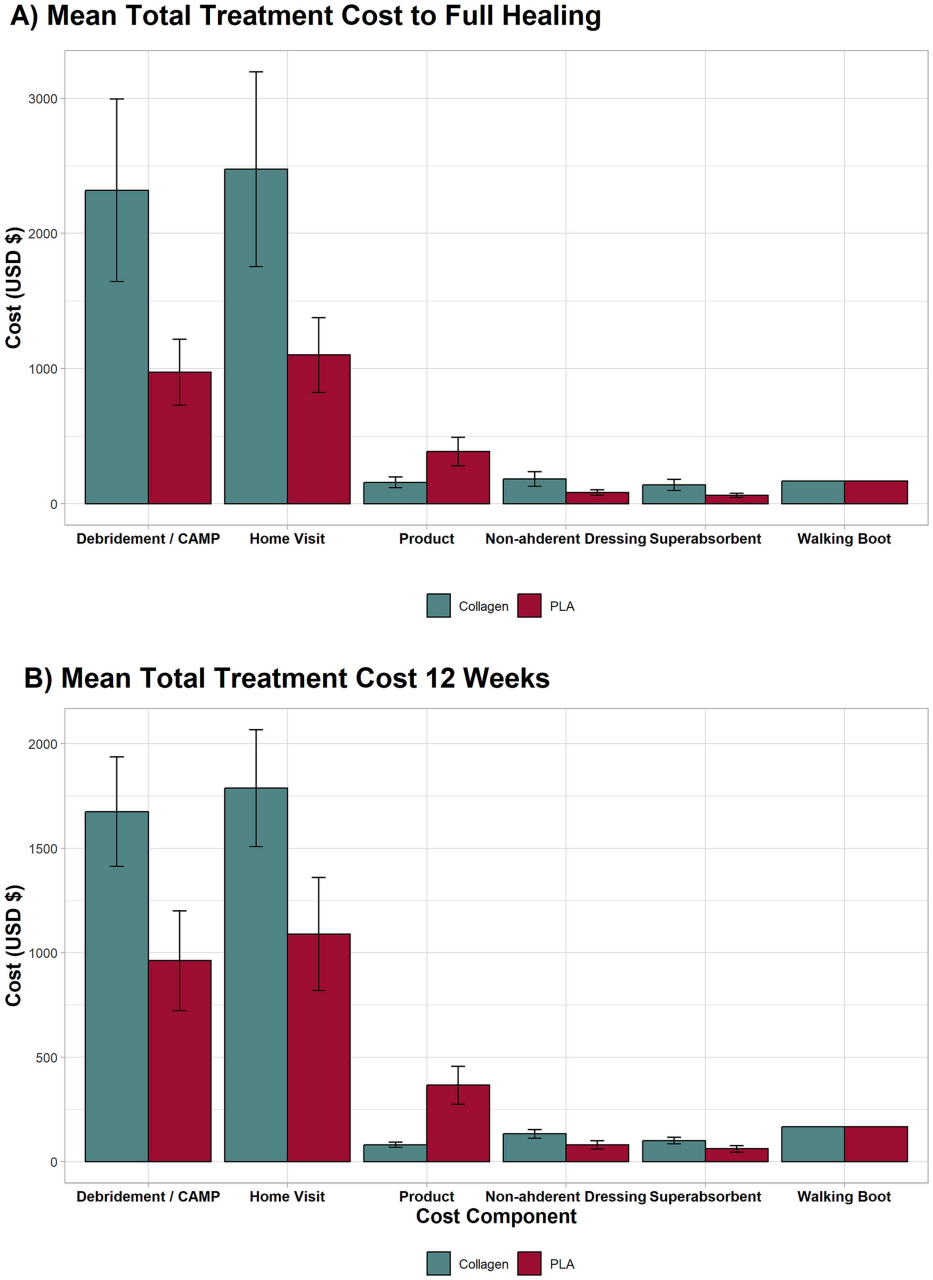
Breakdown of major cost components for the full length of the trial and 12 weeks. The highest contributors to costs were debridement, wound care, and home visits. Even without home visit costs, the collagen group remained more expensive due to longer treatment duration.

Figure 2 provides a detailed breakdown of the individual cost components for both treatments at week 12 and full healing. The most significant cost drivers in both groups at week 12 and full healing were debridement (week 12: PLA group mean cost = $962, collagen group mean cost = $1,675, mean difference $713 95% CI 375 to 1,051, *p* < 0.001; full healing: PLA group mean cost = $972, collagen group mean cost = $2,318, mean difference $1,346 95% CI 660 to 2,031, *p* < 0.001) and home healthcare visits (week 12: PLA group mean cost = $1,089, collagen group mean cost = $1,787, mean difference $698 95% CI 327 to 1,069, *p* < 0.001; full healing: PLA group mean cost = $1,101, collagen group mean cost = $2,474, mean difference $ 1,373 95% CI 636 to 2,109, *p* < 0.001). This costs were followed by product (collagen dressings or PLA; week 12: PLA group mean cost = $366, collagen group mean cost = $80.53, mean difference $286 95% CI 198 to 373, *p* < 0.001; full healing: PLA group mean cost = $386, collagen group mean cost = $159, mean difference $228 95% CI 120 to 336, *p* < 0.001); non-adherent dressings (week 12: PLA group mean cost = $80, collagen group mean cost = $133, mean difference $52 95% CI 24 to 79, *p* < 0.001; full healing: PLA group mean cost = $82, collagen group mean cost = $184, mean difference $102 95% CI 47 to 157, *p* < 0.001); super absorbent dressings (week 12: PLA group mean cost = $61, collagen group mean cost = $101, mean difference $39 95% CI 18 to 60, *p* < 0.001; full healing: PLA group mean cost = $62, collagen group mean cost = $139, mean difference $77 95% CI 36 to 119, *p* < 0.001); and walking boot (one-time fixed cost of $167).

**Figure 3:**
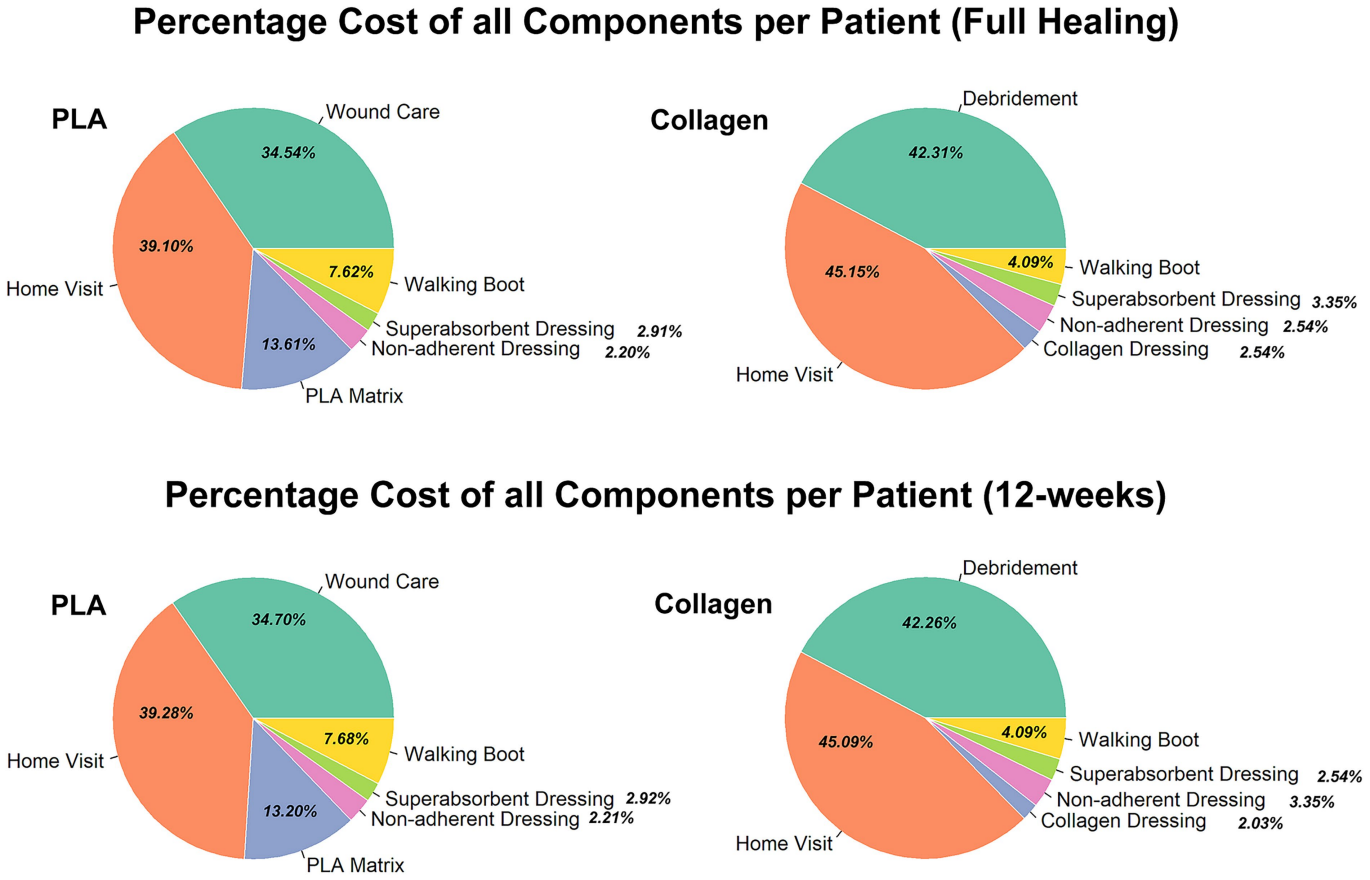
Percentage of all cost components per patient (full healing 12-week) for each group. Comparison of the percentage breakdown of costs for the collagen and PLA groups.

Despite these costs, the total expenditure for PLA was substantially lower, as the product costs accounted for less than 15% of the total as exhibited in Figure 3. In contrast, product costs for the collagen group represented approximately 2.5% of total expenditures, with the overwhelming majority of costs arising from prolonged treatment, particularly home healthcare and ongoing wound care. This finding underscores that duration of care, rather than product price, is the primary driver of total costs. A sensitivity analysis further demonstrated that even when home healthcare costs were excluded from the cost model, the PLA group still had lower total treatment costs than the collagen group.

### 3.2 Cost effectiveness with material usage rounded up to account for wastage

#### 3.2.1 Treatment cost over time

**Figure 4:**
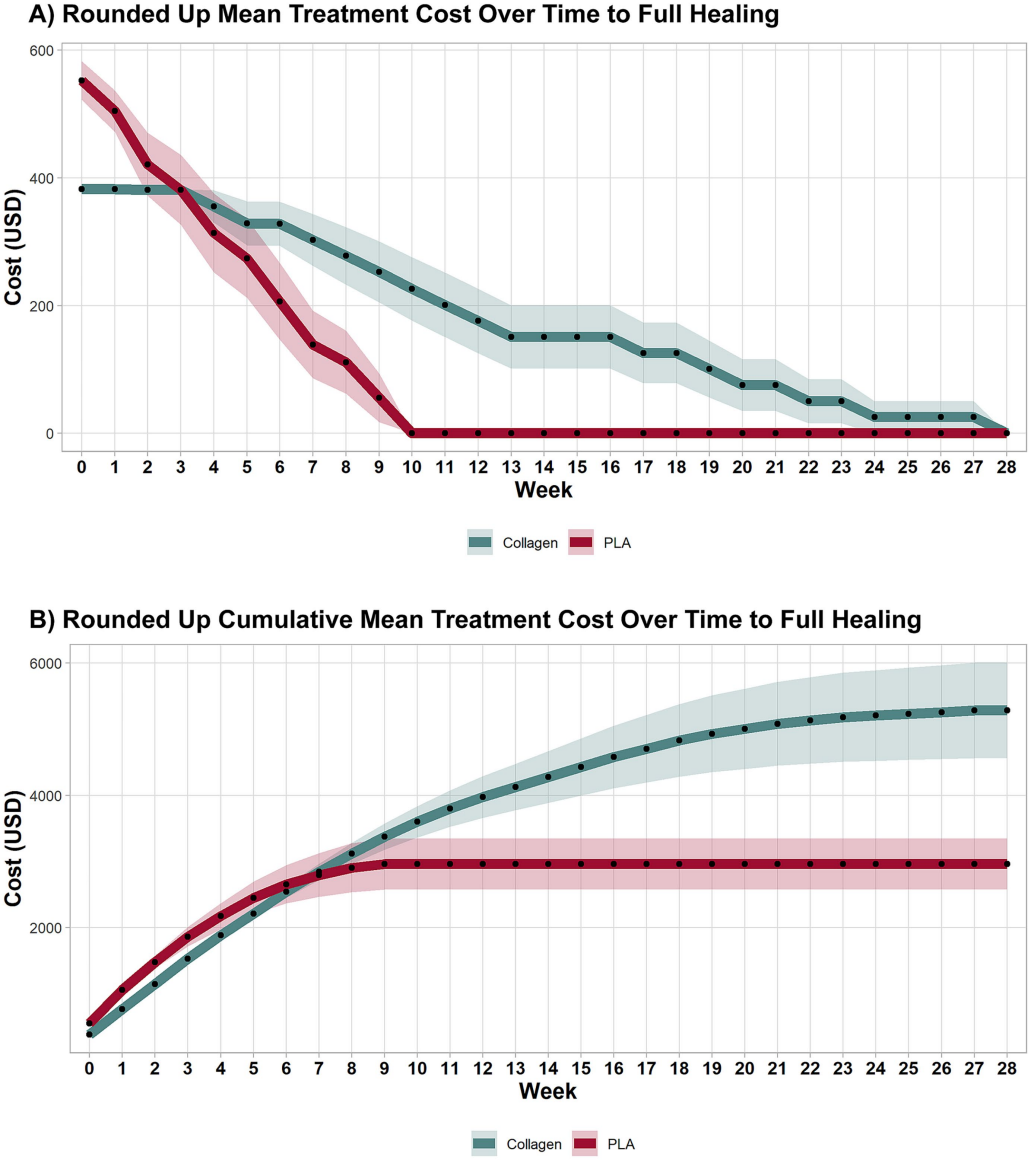
Weekly and cumulative costs until full-healing time points under sensitivity analysis. A sensitivity analysis explored costs accounting for wastage. As in the previous analyses, collagen treatment remained more expensive than PLA. Shaded areas represent the 95% CI.

When material usage was rounded up to account for wastage, the treatment cost over time remained consistent with the first scenario. As shown in Figure 4A, PLA-treated patients incurred no additional costs after week 10, while collagen-treated patients continued to require treatment and accrued costs until week 28. This result aligns with the faster healing times associated with PLA.

#### 3.2.2 Cumulative treatment costs

The cumulative costs for both groups in this scenario are shown in Figure 4B. The inclusion of wastage did not significantly alter the overall cost dynamics, with the collagen group continuing to accumulate higher costs than the PLA group. By the end of the study, total mean costs for collagen were nearly twice those of PLA, consistent with the first scenario. Specifically, the cost to achieve wound closure was $5,284 for collagen and $2,989 for PLA, mean difference $2,295 (95% CI 617.30 to 3,972.00, *p* < 0.001), highlighting the cost-effectiveness of PLA even when factoring in material wastage.

#### 3.2.3 Costs at 12 weeks

As illustrated in Figure 4B, PLA patients had no additional costs beyond the 12-week mark, while collagen patients continued to incur costs until the 28th week. This difference in treatment duration underscores the cost-efficiency of PLA. The cost at week 12 was $3,976 for collagen and $2,961 for PLA, with a mean difference of $1,015 (95% CI 109.08 to 2,032, *p* = 0.041). A sensitivity analysis excluding home visit costs further confirmed that collagen treatment remained more expensive than PLA, even when wastage was accounted for.

### 3.3 Health outcomes

Health outcomes were calculated using QALYs, which combine the impact of quality of life (utility) and quantity of life (time) associated with our intervention. Results show that health outcomes were superior in the PLA group. PLA-treated patients experienced approximately 0.46 QALY, equivalent to 24 weeks of full health, compared to 0.30 QALY, equivalent to 20 weeks in the collagen group. The difference of 0.16 QALY therefore translates into an additional 4 weeks of full health for patients treated with PLA.

## 4 Discussion

This study provides strong evidence supporting the clinical efficacy and cost-effectiveness of PLA dermal matrices compared to collagen-based dressings in the management of DFUs. The analysis demonstrates that the PLA group consistently achieved superior health outcomes while simultaneously incurring in lower costs. The key finding that PLA-treated patients experienced faster wound closure compared to those treated with collagen highlights the clinical benefits of this advanced wound care therapy.

The cost-effectiveness of PLA was evident throughout the study. Patients in the PLA group had a significantly lower overall treatment cost (mean cost 43% lower) and experienced better health outcomes, as indicated by a higher QALY score compared to the collagen group (extra 4 weeks of complete health). This highlights a clinically meaningful advantage in quality of life associated with PLA use. The higher QALY score in the PLA group reflects not only faster healing but also the improved quality of life that results from a shorter treatment duration. By reducing the time patients spend in an unhealed state, PLA helps minimize the physical and emotional burden associated with chronic wounds, leading to a better overall patient experience.

One critical insight from the analysis is that home healthcare and wound care services were the major cost drivers in both the PLA and collagen groups, which reflect the duration and intensity of treatment required, rather than the cost of the products themselves. This finding is important for understanding the broader economic implications of DFU treatments. Although PLA matrices have a higher upfront cost than collagen dressings, the shorter treatment duration and faster healing times associated with PLA reduce the need for prolonged home care and wound management. The clinical implication of this is that by accelerating wound closure, PLA not only reduces direct product-related expenditures but also substantially decreases the need for supportive services. In practice, this translates into fewer nursing visits, less prolonged patient dependence on healthcare resources, and lower risk of complications associated with delayed healing. Therefore, interventions such as PLA that shorten time-to-heal offer profound benefits that extend well beyond the initial product cost, aligning both economic and patient-centered outcomes. By week 12, most patients in the PLA group had achieved full wound closure, whereas patients in the collagen group continued to incur additional costs for up to 28 weeks. Therefore, despite the higher upfront cost of PLA matrices, their ability to expedite healing reduces the need for prolonged care, frequent medical interventions, and the associated healthcare costs.

Sensitivity analyses further demonstrated that even when home healthcare costs were excluded from the cost model or when accounting for product wastage, the PLA group still had lower total treatment costs than the collagen group. This robustness in cost savings highlights the economic advantage of PLA matrices, even when certain cost factors are removed. The sensitivity analyses also emphasize that PLA’s cost-effectiveness remains valid across various healthcare settings and scenarios.

The clinical advantages of PLA go beyond just cost savings. By significantly reducing the time to wound closure, PLA improves patients’ quality of life by allowing them to return to normal activities more quickly. Chronic wounds, such as DFUs, are associated with high morbidity and prolonged healing times, which can lead to complications like infections or amputations and increase healthcare costs. The faster healing times observed in the PLA group reduce these risks, offering a dual benefit of improved clinical outcomes and lower healthcare expenditures. From a healthcare system perspective, the wide adoption of PLA matrices could help reduce the economic burden of chronic wound care. For example, it is estimated that at least one-third of people with diabetes will develop a foot ulcer during their lifetime, and that DFUs annually affect about 18.6 million people worldwide and 1.6 million in the US alone (3). Therefore, using the cost saving per patient found in this study of $2,295 multiplied by 1.6 million of ulcers, would lead to a reduction of approximately $3.7 billion annually, representing a substantial reduction in the estimated US chronic wound care expenditure of $9 to 13 billion (1, 5). By improving clinical outcomes and shortening the duration of treatment, PLA not only benefits individual patients but also contributes to the more efficient use of healthcare resources.

It is important to recognize that DFU management does not follow a “one size fits all” approach. Rather, it encompasses a broad spectrum of disease severity, ranging from superficial, uncomplicated ulcers to deep, infected, and ischemic wounds requiring advanced interventions. This heterogeneity influences both clinical outcomes and economic impact, limiting the extent to which results from a single study can be generalized. However, when compared to published data, the cost benefits of PLA are still clear. CAMPs are effective in treating DFUs, with some studies indicating potential cost-effectiveness (19, 20). However, the cost-effectiveness of CTPs varies widely, with healing rates ranging from 28 to 68% and treatment costs per DFU in the office setting, ranging from $1,207 to $8,791 (21). With a mean total cost of $2,989 for healing a DFU, PLA is in the lower end of this spectrum, with the much higher advantage of having a healing rate of 80 to 85% (11, 12). Furthermore, when compared to amnion-based products, which have a mean clinical effectiveness of 50% and a mean 12-week cost of $4,149 (22), at $2,961 for a similar period, PLA is 33% less costly. Furthermore, when compared to newer generation CAMPs, such as fish skin grafts, PLA has a much greater cost-effectiveness. The total cost for healing a DFU using fish skin grafts was estimated as $7,364.05 (7), which is 146.4% higher than PLA (Table 5).

**Table 5:** Comparative effectiveness and cost of selected dressings and CAMPs in DFU management.

CAMP/CTP	Healing rate (12 weeks)	Reported cost (USD) per episode	% Difference cost vs PLA	Citation
PLA Matrix	~80%	~ $2,500	NA	(11, 12)
Intact Fish Skin Graft	~60%	~$7,000	+146%	(7)
Human Amniotic Membrane	~50%	~$4,000	+33%	(22)
Collagen Dressings	~30%	~$4,000	+33%	(11)

In summary, the use of PLA dermal matrices represents a cost-effective and clinically superior alternative to traditional collagen dressings for DFU management. The faster healing times associated with PLA improve the quality of life for patients, reduce the duration of treatment, and decrease the overall cost of care. These findings highlight the potential of PLA to optimize both clinical outcomes and healthcare resource utilization, supporting its broader adoption as a treatment for DFUs.

### 4.1 Limitations

While this study provides robust evidence supporting the cost-effectiveness and clinical benefits of PLA dermal matrices in the treatment of DFUs, several key limitations should be considered.

The study’s results are based on a single RCT conducted in a specific region with a relatively small sample size. This may limit the generalizability of the findings to other populations and healthcare settings. To ensure broader applicability, future studies should include larger, more diverse patient populations across different geographic locations and healthcare systems. This would allow for a better understanding of how the cost-effectiveness of PLA matrices varies across different demographic and clinical contexts.

A key limitation of our analysis is the 28-week time horizon, which mirrors the duration of the underlying RCT. The time horizon of 28 weeks, while sufficient to capture the primary endpoint of wound closure, does not account for longer-term outcomes such as wound recurrence, reinterventions, or amputation risk, which are highly relevant in the management of diabetic foot ulcers. DFUs are prone to recurrence, and the potential for reoccurrence or the need for additional treatments beyond the trial period could influence the long-term cost-effectiveness of PLA matrices (3). Each recurrent ulcer episode significantly increases the likelihood of severe complications and, from a health economics perspective, these events carry profound cost and quality-of-life consequences. Future studies with extended follow-up are therefore critical to fully capture the long-term value of PLA in reducing not only time-to-heal but also recurrence-related morbidity and economic burden.

As of the moment of writing this research, the US Centers for Medicare & Medicaid Services local coverage determination (LCD) covers up to 12 applications of CAMPs within a 12-week period. Therefore, we considered the 12^th^ week as a relevant time point for our analysis. However, it is expected that new guidelines will come into effect in 2026 that will modify the LCD to up to 8 CAMP applications over a 16-week period (23). When analyzed at the 16^th^ week, our data shows significant cost savings for the use of PLA matrices for treating DFUs (mean difference vs. collagen dressings of $1,947; 95% CI 798 to 3,096; *p* < 0.001). However, the data presented here came from weekly applications and not bi-weekly applications, which is what the LCD guideline implies. Thus, it could be expected that the cost reduction would be even greater for the PLA group should clinical effectiveness remain similar. Nonetheless, research needs to be carried out to support this claim.

The analysis focused solely on direct medical costs, such as debridement, wound care products, and home healthcare visits, but did not consider indirect costs like lost productivity, caregiver burden, or the emotional and social impacts on patients with chronic wounds. These indirect costs have a substantial impact on patients’ lives and the overall economic burden of DFUs (3, 5, 7). Including indirect costs in future studies would provide a more comprehensive understanding of the true economic impact of PLA matrices and other wound care treatments.

Although the study included sensitivity analyses that confirmed PLA’s cost-effectiveness even when home healthcare costs were excluded and wastage considered, healthcare costs can vary significantly across regions and settings. The assumptions made regarding product usage, wastage, and regional variations in healthcare services may not fully reflect real-world practices. Future studies should explore the variability in healthcare costs and product usage patterns in different healthcare settings to better assess the real-world cost-effectiveness of PLA.

Finally, the generalizability of our findings is limited by the single RCT and modest sample size on which the analysis is based. To strengthen future applicability, larger and multi-center studies should prioritize enrolling diverse subsets of DFU patients, particularly those with higher HbA1c levels, advanced age, peripheral arterial disease, or more complex ulcer presentations (e.g., Wagner grade ≥3), as these groups often experience worse outcomes and higher treatment costs. Including such populations will help ensure the applicability of future health economic evaluations across the full spectrum of DFU patients.

## 5 Conclusion

The use of PLA dermal matrices represents a cost-effective and clinically superior alternative to collagen-based dressings for the treatment of DFUs. Faster healing times and reduced healthcare costs make PLA a valuable option for DFU management. The results of this study provide strong clinical and financial evidence supporting the use of PLA matrices in the management of DFUs in appropriately selected patients. Further studies are needed to validate these findings in broader, more complex patient populations before widespread adoption can be recommended. Nevertheless, PLA matrices should be considered a key tool in the effort to improve patient outcomes and reduce the economic burden associated with chronic wound care.

## Data Availability

The raw data supporting the conclusions of this article will be made available by the authors, without undue reservation.
